# Endoscopic sinus surgery dissection courses using a real simulator: the benefits of this training^☆^^☆☆^

**DOI:** 10.1016/j.bjorl.2015.02.003

**Published:** 2015-10-19

**Authors:** Bibiana Fortes, Leonardo Balsalobre, Raimar Weber, Raquel Stamm, Aldo Stamm, Fernando Oto, Nathália Coronel

**Affiliations:** Hospital Professor Edmundo Vasconcelos (HPEV), São Paulo, SP, Brazil

**Keywords:** Nasal cavity, Dissection, Training courses, Cavidade nasal, Dissecação, Cursos de capacitação

## Abstract

**Introduction:**

Endonasal surgeries are among the most common procedures performed in otolaryngology. Due to difficulty in cadaver acquisition and the intrinsic risks of training residents during operations on real patients, nasosinusal endoscopic dissection courses utilizing real simulators, such as the Sinus Model Otorhino Neuro Trainer are being developed as a new technique to facilitate the acquisition of better anatomical knowledge and surgical skill.

**Objective:**

To evaluate the efficacy of nasosinusal endoscopic dissection courses with the Sinus Model Otorhino Neuro Trainer simulator in the training of otolaryngology surgeons.

**Methods:**

A prospective, longitudinal cohort study was conducted with 111 otolaryngologists who participated in a theoretical and practical course of endoscopic surgery dissection using the Sinus Model Otorhino Neuro Trainer simulator, with application of questionnaires during and after the course.

**Results:**

From the ten procedures performed utilizing the simulator, the evaluation revealed mean scores from 3.1 to 4.1 (maximum of 5). Seventy-seven participants answered the questionnaire six months after the end of the course. 93% of them reported that they could perform the procedures more safely following the course, 98% reported an improvement in their anatomical and clinical knowledge, and 85% related an improvement in their surgical ability. After the course, the number of endoscopic surgeries increased in 40% of the respondents.

**Conclusion:**

Endoscopic sinus dissection courses using the Sinus Model Otorhino Neuro Trainer simulator proved to be useful in the training of otolaryngologists.

## Resumo

**Introdução:**

Cirurgias endonasais são as cirurgias mais realizadas na otorrinolaringologia. Devido à dificuldade de adquirir cadáveres e ao risco inerente em realizar treinamento em pacientes reais, cursos de dissecção endoscópica nasossinusal em simuladores reais, como o sinus model otorhino – neuro trainer – S.I.M.O.N.T, são uma nova proposta de aquisição de maior conhecimento anatômico e habilidades cirúrgicas.

**Objetivo:**

Avaliar a eficácia dos cursos de dissecção de cirurgia endoscópica nasossinusal com simulador S.I.M.O.N.T. no treinamento de cirurgiões otorrinolaringologistas.

**Método:**

Estudo de coorte contemporâneo longitudinal com 111 otorrinolaringologistas que participaram de curso teórico – prático de dissecção endoscópica nasossinusal em simulador real S.I.M.O.N.T., com aplicação de questionários durante e após a realização do curso.

**Resultados:**

Dos dez procedimentos realizados no simulador, a avaliação apontou para médias de notas que variaram de 3,1 a 4,1 (máximo de 5). Setenta e sete participantes responderam a avaliação 6 meses após o curso. Declararam que houve aumento da segurança após o curso, 93% dos mesmos; 98% disseram ter aumentado o conhecimento anátomo-clinico e 85% declararam um aumento da habilidade cirúrgica. Quarenta por cento aumentaram o número de cirurgias endonasais após o curso.

**Conclusão:**

Cursos de dissecção de cirurgia endonasais com simulador real S.I.M.O.N.T. mostraram ser úteis para o treinamento de otorrinolaringologistas.

## Introduction

Endonasal surgeries are among the most performed surgeries in otolaryngology. They are considered the standard of treatment of several sinus and nasal cavity pathologies.^1^, ^2^ Due to their importance, resident training is a constant concern in medical residency services. The manipulation of anatomical structures and surgical instruments during the procedure is challenging for inexperienced surgeons, due to the complex anatomy of intranasal region and the intimate relationship with vital structures, such as the brain, carotid artery, and orbit components.^1^, ^2^, ^3^, ^4^, ^5^

Currently, most training in endonasal surgery is conducted in the operating room on real patients under the supervision of more experienced surgeons.^2^, ^4^, ^5^, ^6^ Since approximately 5–10% of endonasal surgeries develop complications and since the learning curve for performing surgical procedures on real patients may add an additional risk, it would be ideal to minimize this risk as much as possible. Thus, activities in dissection labs or in simulators, in addition to theoretical and practical courses of dissection, should be performed and encouraged. However, the development of such laboratories has some challenges, including ethical, legal, financial and technical problems that hinder the acquisition of cadavers or animal models; therefore the surgical training process in otolaryngology is gradually moving toward to the use of surgical simulators.^2^, ^4^, ^5^, ^6^, ^7^

Surgical simulators can be divided into two types: virtual simulators and real anatomical models.^3^ Virtual simulators are based on interactive computer programs, *i.e.*, they use virtual reality elements and mechanisms of direct interaction with users.^3^, ^4^, ^5^ Conversely, the Sinus Model Otorhino Neuro Trainer (S.I.M.O.N.T.), a real anatomical model, was created from images of anatomical structures, computed tomography, and endoscopic anatomical dissection videos of cadavers, providing training, by dissection, of endoscopic nasosinusal surgery.^3^, ^4^, ^8^

In this service, the simulator adopted for training during the otolaryngologists’ training courses is the S.I.M.O.N.T., a real simulator.

Training courses in nasosinusal surgery on cadavers provide unequivocal benefit to surgeons,^9^ but studies demonstrating equivalent results in courses with real simulators are still needed.

## Objective

The purpose of this study was to evaluate the effectiveness of dissection courses for endoscopic nasosinusal surgery, using the S.I.M.O.N.T. simulator to train otolaryngology surgeons.

## Methods

This research was conducted through a study of a longitudinal contemporary cohort. Three questionnaires were applied to otolaryngologists who took part in practical training courses in endoscopic nasosinusal surgery with the simulator in the years 2011 and 2012. The course features theoretical lectures on anatomy as well as surgical and dissection technique concepts in a model.

**Figure 1:**
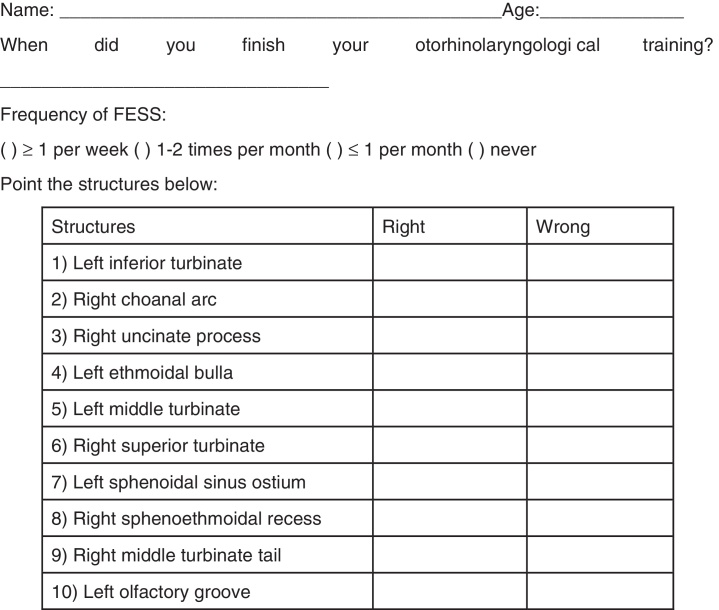
Questionnaire 1.

The first questionnaire (Fig. 1) was applied after the theoretical lectures and before dissection with S.I.M.O.N.T. simulator and aimed to the basic knowledge of endonasal anatomy of participants, by identifying ten anatomical structures.

**Figure 2:**
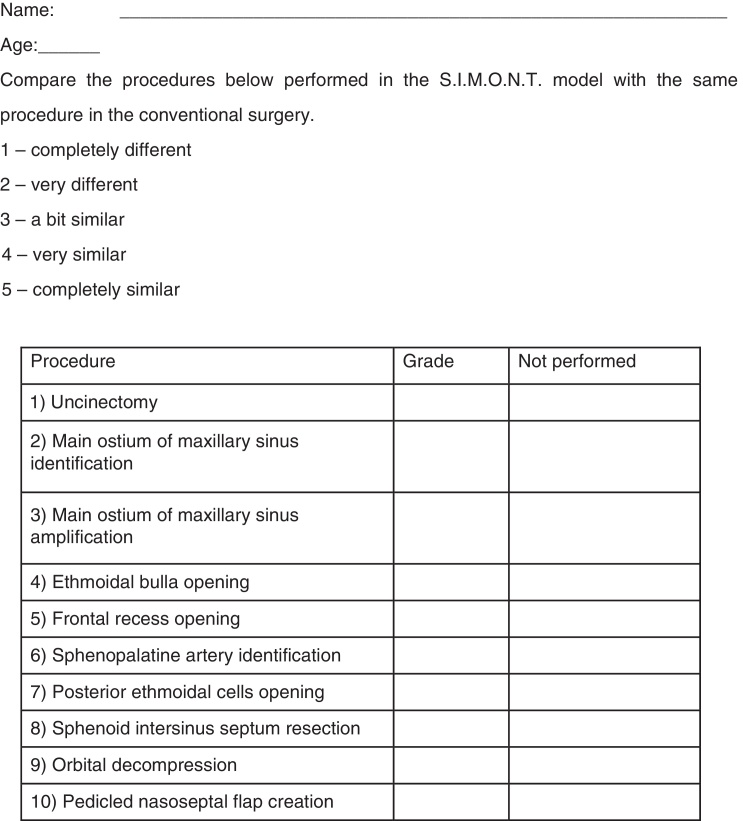
Questionnaire 2.

The second questionnaire (Fig. 2) aimed to assess how closely the dissection in the model approximated reality, and was applied after dissection and theoretical lectures. Participants were required to assign grades (from 1 to 5) comparing ten procedures performed employing endonasal surgery in a human being or in a cadaver to the same procedure performed during dissection of the S.I.M.O.N.T.; the subjects assigned a value of 0 when the procedure was not performed, 1 when the procedure was considered as completely different, 2 for very different, 3 for somewhat similar, 4 for very similar, and 5 for a completely similar procedure.

**Figure 3:**
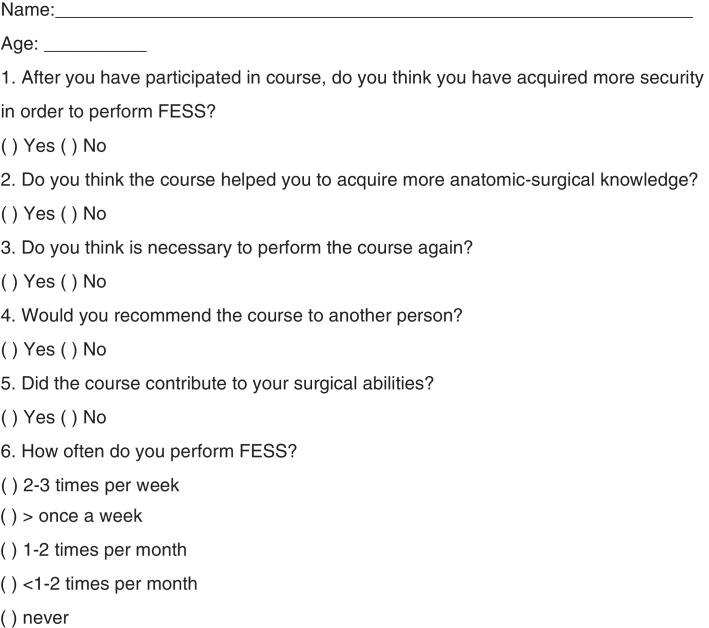
Questionnaire 3.

The third and final questionnaire (Fig. 3) was obtained by telephone or by e-mail at least six months after the course, to assess the impact of the training with the simulator in the participant's medical practice.

Participants were divided into two groups according to the surgical experience at the time of dissection with the S.I.M.O.N.T. Group A (more experienced trainees) consisted of surgeons who performed at least one endonasal surgery per week and those who performed once or twice a month. Group B (less experienced trainees) comprised participants who performed less than one surgery per month and those who never had performed an endonasal surgery.

## Results

The study involved the participation of 111 surgeons. Sixty (54.1%) of these participants were otolaryngology residents; 51 (45.9%) otolaryngologists who had already graduated with a mean of 5.9 years of practice in the specialty, ranging from one to 23 years. Fifty-one surgeons (45.9%) who performed endonasal surgery more than once per week and 36 (32.4%) who performed the surgery two to three times per month were classified as Group A. The other 18 (16.2%) participants who performed less than one monthly procedure and six (5.4%) who had never performed endonasal surgery were classified as Group B.

### Questionnaire 1

The mean of the correctly identified anatomical structure for Group A was 88.4%, and for Group B was 86.2%. The anatomical structures most commonly mis-identified were the superior turbinate, olfactory groove, and sphenoethmoidal recess (Table 1).

**Table 1:** Percentiles of correct answers about anatomic structures.

Structures	Group 1 (87)	Group 2 (24)
	*n* (%)	*n* (%)
Left inferior turbinate	87 (100%)	24 (100%)
Right choanal arc	81 (93.1%)	24 (100%)
Right uncinate process	70 (80.5%)	18 (75%)
Left ethmoidal bulla	84 (96.6%)	22 (91.6%)
Left middle turbinate	86 (98.9%)	23 (95.3%)
Right superior turbinate	64 (73.6%)	13 (54.2%)
Left sphenoidal sinus ostium	79 (90.8%)	21 (87.5%)
Right sphenoethmoidal recess	67 (77%)	20 (83.3%)
Right middle turbinate tail	85 (97.7%)	24 (100%)
Left olfactory groove	66 (75.9%)	18 (75%)
Median	88.41%	86.19%

### Questionnaire 2

**Figure 4:**
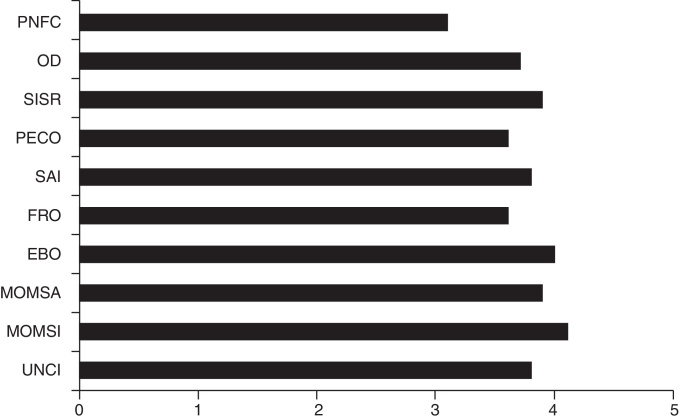
Mean grades attributed to the procedures. PNFC, pedicled nasoseptal flap creation; OD, orbital decompression; SISR, sphenoid intersinus septum resection; PECO, posterior ethmoidal cells opening; SAI, sphenopalatine artery identification; FRO, frontal recess opening; EBO, ethmoidal bulla opening; MOMSA, main ostium of maxillary sinus amplification; MOMSI, main ostium of maxillary sinus identification; UNCI, uncinectomy.

Of the ten procedures that were performed in the simulator, the evaluation of surgeons resulted in mean scores ranging from 3.1 to 4.1 (minimum of 1 and maximum of 5). Procedures that received the lowest scores were pedicled nasoseptal flap creation (mean score 3.1), frontal recess opening (mean score 3.6), and posterior ethmoid bulla opening (mean score 3.6). The procedures less performed were resection of the intersinusal sphenoid septum, orbital decompression, and pedicled nasoseptal flap creation (Fig. 4).

### Questionnaire 3

Of the 111 initial participants, 77 answered the questionnaire at least six months after completing the course. Table 2 demonstrates the percentages of each response.

**Table 2:** Percentages of answers from Questionnaire 3.

Questions	Yes	No
… have acquired more security in order to perform FESS?	72 (93.5%)	5 (6.5%)
… have acquired more anatomic-surgical knowledge?	76 (98.7%)	1 (1.3%)
… is necessary to perform the course again?	33 (42.8%)	44 (57.2%)
… would you recommend the course to another person?	75 (97.4%)	2 (2.6%)
… contribute to your surgical abilities?	66 (85.7%)	11 (14.3%)

**Figure 5:**
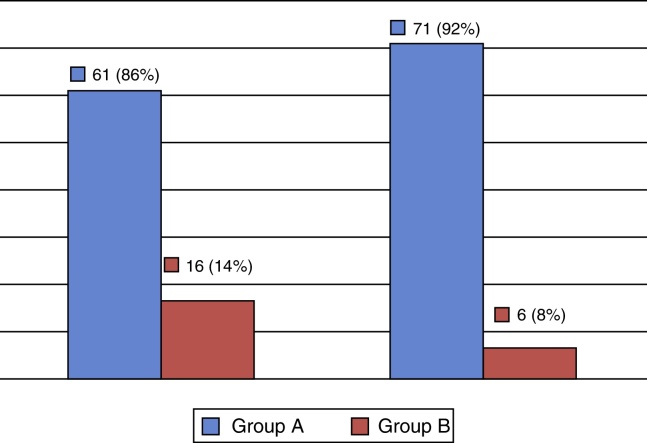
Number of surgeons before and after S.I.M.O.N.T dissection course, according to experience groups.

The last question of the questionnaire refers to the frequency of surgeries after the course. In thirty participants (38.9%) the number of surgeries increased, while in 47 (61.1%) frequency of surgery maintained the same. When stratified into Group A (more experienced) and Group B (less experienced), Fig. 5 shows an increase of 6% in the more experienced group.

## Discussion

S.I.M.O.N.T. presents many advantages when compared with animal or cadaveric models, or with virtual simulators: there is no need of a special place or technique for its storage; it is easily cleaned after use; it uses the same surgical instruments as used in practice; it has the capability to simulate and represent different diseases; it lacks the biological risks inherent in traditional dissections of fresh cadavers; and it can be used to dissect and train in almost any available space. This anatomical model allows training for numerous procedures during the dissection courses.^3^, ^4^, ^8^, ^10^, ^11^

Cadaveric dissection courses are considered extremely important in the education, training, and qualification of ENT surgeons, especially in endoscopic nasosinusal surgery. Gurr et al. showed that 72% of participants in three dissection courses classified this type of training as good or very good, and that the courses currently offered in this area are beneficial and effective.^12^ Braun et al., after a multicenter study evaluating 133 otolaryngologists who attended courses of dissection in cadavers, reported that participants with and without experience in endonasal surgery reported the acquisition of more anatomical knowledge, greater surgical skill, and increased confidence to perform such surgery.^12^

This study aimed to evaluate the S.I.M.O.N.T. simulator in dissection courses of endoscopic nasosinusal surgery, because it is difficult in Brazil to obtain cadavers for achieving such training. This is the first Brazilian study evaluating the role of these courses in a real simulator in a scenario of otolaryngologist training.

The first part of the study, involving Questionnaire 1, applied after theoretical lectures on anatomy and surgical technique and prior to dissection, showed that both Group A and Group B, achieved a high rate of correct identification of anatomical structures. This suggests that, in addition to the similarity between the anatomy of the S.I.M.O.N.T. simulator and the human anatomy (Fig. 2), there was also consolidation of information regarding the anatomical knowledge provided in the theoretical part of the course. Thus, the need for theoretical classes in dissection courses can be inferred.^12^ With regard to those structures with higher error rate: right superior turbinate (RST), left olfactory groove (LOG), and right sphenoethmoidal recess (RSER), the instructors of the course (surgeons with extensive experience) were asked to conduct an investigation on this result. As to RST, it was found that this structure was located more inferiorly than where it is usually found in the body. Regarding LOG and RSER, it is believed that the reason for the low percentage of success may be due to a lack of knowledge or experience on the part of the participants.

The mean grades of the procedures performed in the simulator (Questionnaire 2) ranged from 3.1 to 4.1, for a total of 5 points, suggesting that a dissection course using the S.I.M.O.N.T. allows good training, and is comparable to a course of dissection in a cadaver.

Pedicled nasoseptal flap creation (PNFC), frontal recess opening (FROp), and posterior ethmoidal cell opening (PECBp) procedures obtained the lowest scores, probably because these are considered technically more complex, are not common, and also take longer to perform. Similar findings were described in cadaver dissection courses, where the surgeries involving the posterior ethmoid, the sphenoid, and the frontal sinus were elected as the most difficult procedures to be carried out.^12^ This situation was also confirmed in a study published by the otolaryngology department of the Johns Hopkins Hospital, where the expectation of residents at the end of their training was only to acquire confidence in performing maxillary antrostomies and anterior ethmoidectomies.^13^

The results in Table 2 show a significant increase in safety, anatomical and surgical knowledge, and surgical skill after the course; thus, it can be concluded that training in a real simulator is satisfactory and effective, especially when augmented by theoretical instruction. About 43% of participants reported that they felt the need to take the course again, suggesting that even after completion of training, the acquisition of knowledge is a great challenge; and that maybe only a course is not enough to provide confidence to the surgeon. Yet 97% of otolaryngologists would recommend the course, reinforcing the purposes of the endoscopic nasosinusal surgery dissection course.

Finally, after the course, when participants were asked if there was an increase in the number of their surgeries, about 40% answered affirmatively; and when the subjects were stratified by experience group, there was an increase from 86% to 92% in the group of the most experienced surgeons, and consequently a decrease in those considered less experienced. This supports the idea that surgeons were putting into practice the knowledge and skills acquired in the course, increasing their frequency of surgeries. Thus, despite the good results observed in this study, a constant improvement in the quality of anatomy and of the material used in the simulator should be maintained, in order to optimally correspond to a cadaveric experience.

## Conclusion

It was demonstrated that nasosinusal endoscopic surgical dissection courses with the S.I.M.O.N.T., a real simulator, are useful and beneficial for the training and qualification of otolaryngology surgeons.

## Conflicts of interest

The authors declare no conflicts of interest.
